# Long-term oncological outcomes of laparoscopic versus transanal total mesorectal excision for mid-low rectal cancer: a propensity score matching analysis

**DOI:** 10.3389/fonc.2026.1715774

**Published:** 2026-02-05

**Authors:** Yang Xie, Ziwei Wang, Hongyu Zhang

**Affiliations:** 1 Department of Gastrointestinal Surgery, The First Affiliated Hospital of Chongqing Medical University, Chongqing, China; 2 Department of General Surgery, Chongqing General Hospital, Chongqing University, Chongqing, China

**Keywords:** oncological outcomes, transanal total mesorectal excision, rectal cancer, laparoscopic surgery, overall survival

## Abstract

**Background:**

Transanal total mesorectal excision (taTME) has become a promising surgical approach for anus-preserving surgery of mid-low rectal cancer (RC). This study aimed to compare the long-term oncological outcomes of taTME and laparoscopic total mesorectal excision (lapTME).

**Methods:**

Of 233 patients who were treated for mid-low RC from July 2017 to August 2020, 110 underwent taTME and 123 received lapTME. Propensity score matching (PSM) was performed to balance the baseline characteristics between the taTME and lapTME groups. After PSM, 61 patients were included in each group.

**Results:**

Prior to PSM, the 5-year overall survival (OS) and disease-free survival (DFS) rates were comparable between the taTME and lapTME groups (72.7% *vs*. 69.1%, *p* = 0.617; 72% *vs*. 69%, *p* = 0.576, respectively). After PSM, there was no statistically significant difference in the 5-year OS and DFS rates between groups (64.2% *vs*. 64.4%, *p* = 0.936; 66.1% *vs*. 66.1%, *p* = 0.947, respectively).

**Conclusion:**

As compared to lapTME, taTME achieved comparable oncological safety for patients with mid-low RC.

## Introduction

Colorectal cancer is the third most common cancer globally in terms of incidence (new cases) and the second most common cause of cancer-related death worldwide (1). Current treatment methods for rectal cancer (RC) include chemotherapy, radiotherapy, surgery, and immunotherapy, with surgery playing a dominant role. Total mesorectal excision (TME) for RC surgery was first proposed in 1982 and has since become the gold standard for radical resection of RC, owing to the capacity to reduce local recurrence (LR) and improve survival (2, 3). Laparoscopic total mesorectal excision (lapTME) has emerged in response to the growing demand for minimally invasive surgery for RC. Some studies have confirmed that the short- and long-term outcomes of lapTME are comparable to those of traditional open surgery (4–6). Additional advantages of lapTME include less postoperative pain, faster recovery, and shorter hospital stays (7). Nonetheless, lapTME remains challenging for obese patients and those with benign prostatic hyperplasia or a narrow pelvis.

A novel “bottom-to-up” surgical approach, known as transanal total mesorectal excision (taTME), was first introduced in 2010 (8). Unlike traditional lapTME, taTME provides improved visibility and a larger surgical field for patients with a narrow pelvis, allowing for a more satisfactory distal resection margin (DRM) and an increased likelihood of anus preservation. Regretfully, due to multifocal LR, the Norwegian colorectal surgical community has suspended taTME surgery (9). In other countries, taTME has gained popularity, with some studies confirming comparable perioperative recovery and short-term oncological outcomes to lapTME (10–12). However, the long-term oncological results of taTME remain controversial. Moreover, appropriate case selection, adequate training, and high-quality surgical collaboration are essential to avoid adverse events associated with taTME (13).

Over the past 10 years, taTME, as a new surgical technique, has faced significant challenges due to the lack of high-quality research on long-term oncological outcomes as a prerequisite for widespread acceptance. The TaLaR Chinese trial demonstrated comparable short-term outcomes between taTME and lapTME, and safe performance by experienced surgeons with a double team (14). Nevertheless, the results of the international multicenter randomized controlled trial (RCT) COLOR III (15) have not yet been published. Therefore, the aim of this retrospective study was to compare the long-term oncological outcomes of taTME and lapTME.

## Materials and methods

### Patient selection

The inclusion criteria were (i) rectal adenocarcinoma with a distance between the tumor and anal verge of less than 10 cm, (ii) clinical stages I–III or stage IV with liver metastasis that could be resected synchronously, and (iii) no serious comorbidity, while the exclusion criteria were (i) Miles surgery, (ii) recurrent RC, (iii) emergency surgery, and (iv) palliative treatment. This retrospective study was approved by the Ethics Committee of the First Affiliated Hospital of Chongqing Medical University Hospital (approval no. 20193501). Based on the above criteria, the clinical data of 233 patients treated by the same team from July 2017 to August 2020 were retrospectively analyzed.

**Figure 1:**
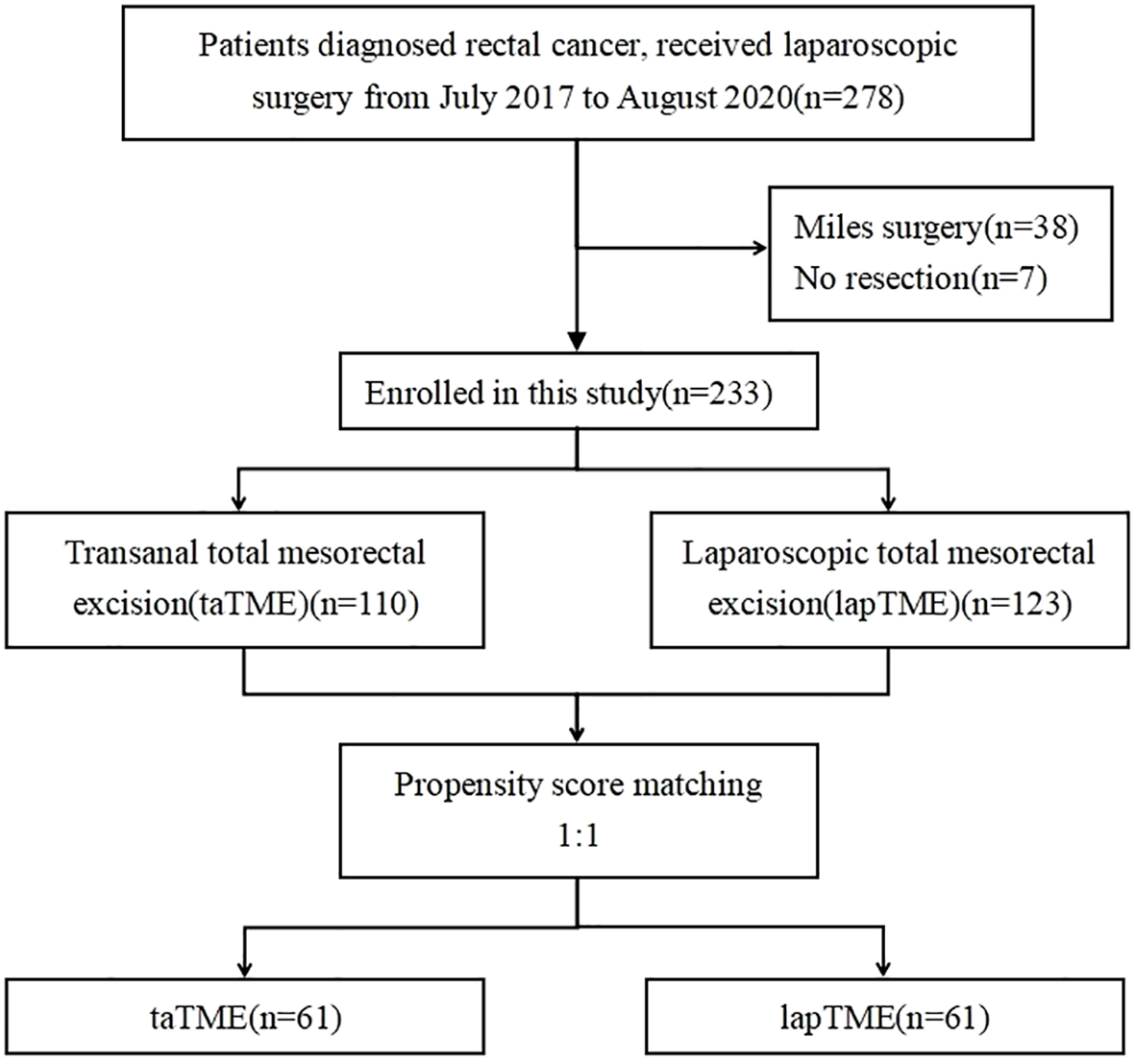
Flow diagram of patients included in the study.

The tumor-node-metastasis (TNM) stage was determined in accordance with the eighth edition of the American Joint Committee on Cancer staging system (16). Patients with locally advanced rectal cancer (cT3–4 or N+), as mostly determined by magnetic resonance imaging (MRI), received neoadjuvant therapy. A flow chart of the patient selection process is presented in **Figure 1**.

### Data collection

The baseline clinical variables, including age, sex, body mass index (BMI), tumor distance from the anal verge, clinical tumor stage, comorbidities, and American Society of Anesthesiologists (ASA) physical status score, and neoadjuvant therapy, were analyzed. The tumor distance from the anal verge was measured by MRI.

Clinicopathological variables included the surgical approach, operative time, estimated blood loss, enterostomy, intraoperative complications, type of anastomosis (handsewn or staple), postoperative complications, TME quality, length between the tumor and DRM, differentiation grade, lymphovascular invasion, nerve invasion, positive rates of circumferential resection margin (CRM) (≤1 mm), numbers of metastatic and total lymph nodes, and pathological tumor stage. Anastomotic leakage was diagnosed based on the criteria of the International Study Group of Rectal Cancer (17). Postoperative complications were graded in accordance with the Clavien–Dindo classification (18). The taTME and lapTME procedures were performed by the same group of experienced surgeons. The decision to create an ileostomy was based on the integrity, blood supply, and tension of the anastomosis.

### Follow-up

Follow-up data were collected through outpatient or telephone consultations. The chief outcomes of the study were overall survival (OS) and disease-free survival (DFS). The secondary outcomes were LR and distant metastases (DM), as determined by histopathological and/or radiological studies.

### Statistical methods

Statistical analyses were performed using IBM SPSS Statistics for Windows (version 27.0; IBM Corporation, Armonk, NY, USA) and R software (version 4.4.3; https://www.r-project.org/). Continuous variables were compared using the *t*-test and are presented as either the mean ± standard deviation or median (interquartile range, IQR). Categorical variables were compared using the χ^2^ test or Fisher’s exact test and are expressed as numbers (percentages). A probability (*p*) value < 0.05 was considered statistically significance. Propensity score matching (PSM) at 1:1 was conducted with a matching tolerance of 0.1 by the nearest neighbor matching method to reduce selection bias of age, sex, BMI, ASA score, comorbidity, tumor stage, neoadjuvant therapy, and tumor distance from the anal verge. The reverse Kaplan-Meier method was used to calculate the median follow-up time. Survival analyses were performed using Kaplan-Meier curves and compared with the log-rank test. Univariate and multivariate analyses were performed using Cox proportional hazard models to identify risk factors affecting OS and DFS. Significant variables (*p* < 0.1) by univariate analysis were included for multivariate analysis. The hazard ratio (HR), *p* value, and 95% confidence interval (CI) were calculated to explain the results.

## Results

### Patient characteristics

Of the 233 patients included in this study, 110 underwent taTME and 123 received lapTME. The basic characteristics of patients before and after PSM are shown in **Table 1**. Before PSM, patients who underwent taTME were mainly male (75.5% *vs*. 58.5%, *p* = 0.006) and younger (59.5 ± 11.7 *vs*. 62.6 ± 11.3, *p* = 0.041), with lower locations of tumors (56.2 ± 13.6 *vs*. 67.7 ± 10.8 mm, *p* < 0.001), lower neoadjuvant therapy rate (29.1% *vs*. 43.1%, *p* = 0.027), and an earlier TMN stage (*p* < 0.001). After PSM, 61 patients were assigned to each group. There was no significant difference in patient characteristics between the two groups.

**Table 1:** Demographics characteristics.

Characteristic	Unmatched cohort			Matched cohort		
	taTME(n=110)	lapTME(n=123)	P	taTME(n=61)	lapTME(n=61)	P
Sex			0.006			0.570
Male, n(%)	83(75.5)	72(58.5)		41(67.2)	38(62.3)	
Female, n(%)	27(24.5)	51(41.5)		20(32.8)	23(37.7)	
Age, mean ± SD	59.5 ± 11.7	62.6 ± 11.3	0.041	60.6 ± 10.6	61.2 ± 11.6	0.789
BMI, mean ± SD	23.2 ± 2.6	23.1 ± 2.2	0.607	23.5 ± 2.7	23.1 ± 2.4	0.440
Tumour distance from anal verge, mean ± SD, mm	56.2 ± 13.6	67.7 ± 10.8	<0.001	62.1 ± 13.5	63.1 ± 10.0	0.653
Clinical TNM stage, n(%)			<0.001			0.213
I	26(23.6)	0 (0)		4(6.6)	0(0)	
II	49(44.5)	40(32.5)		28(45.9)	32(52.5)	
III	33(30.0)	81(65.9)		27(44.3)	28(45.9)	
IV	2(1.8)	2(1.6)		2(3.3)	1(1.6)	
Comorbidity			0.382			0.700
Yes	39(35.5)	37(30.1)		21(34.4)	19(31.1)	
No	71(64.5)	86(69.9)		40(65.6)	42(68.9)	
ASA score, n(%)			0.080			0.951
I	21(19.1)	11(8.9)		8(13.1)	8(13.1)	
II	79(71.8)	100(81.3)		47(77)	48(78.7)	
III	10(9.1)	12(9.8)		6(9.8)	5(8.2)	
Neoadjuvant therapy, n(%)			0.027			0.548
Yes	32(29.1)	53(43.1)		16(26.2)	19(31.1)	
No	78(70.9)	70(56.9)		45(73.8)	42(68.9)	

taTME, transanal total mesorectal excision; lapTME, laparoscopic total mesorectal excision; SD, Standard deviation; BMI, body mass index; TNM, tumour node metastasis; ASA, American Society of Anesthesiologists.

### Perioperative and histopathological outcomes

The perioperative outcomes are shown in **Table 2**. The mean operative time was longer in the TaTME group than the lapTME group (290.6 ± 83.8 *vs*. 243.2 ± 26.1 min, *p* < 0.001), while estimated blood loss was greater [100 (95% CI, 116.2–148.1) *vs*. 100 (95% CI, 90.5–116.1) mL, *p* = 0.006]. More patients in the taTME group underwent enterostomy (39.3% *vs*. 21.3%, *p* = 0.030) and handsewn anastomosis (11.5% *vs*. 0, *p* = 0.013). There were no significant differences in intraoperative complications, postoperative complications, anastomotic leakage, and Clavien-Dindo grade 3+ complications between the two groups. Before PSM, all perioperative outcomes (**Supplementary Table S1**) were similar to the above outcomes.

**Table 2:** Operative details and clinical outcomes of the matched cohort.

Variable	taTME(n=61)	lapTME(n=61)	P value
Operative time, mean ± SD, min	290.6 ± 83.8	243.2 ± 26.1	<0.001
Estimated blood loss, median(95% CI), ml	100(116.2-148.1)	100(90.5-116.1)	0.006
Enterostomy, n (%)			0.030
Yes	24(39.3)	13(21.3)	
No	37(60.7)	48(78.7)	
Intraoperative complications, n (%)	2(3.3)	3(4.9)	0.648
Type of anastomosis, n (%)			0.013
Stapled	54(88.5)	61(100)	
Handsewn	7(11.5)	0(0)	
Postoperative complications, n (%)	14(23.0)	11(18.0)	0.501
Anastomoticleak, n (%)	5(8.2)	5(8.2)	1.000
Clavien-Dindo grade 3+ complications, n (%)	2(3.3)	2(3.3)	1.000

taTME, transanal total mesorectal excision; lapTME, laparoscopic total mesorectal excision; SD, standard deviation; CI, confidence intervals.

The histopathological outcomes are presented in **Tables 3**; **Supplementary Table S2**. Both before and after PSM, there was no significant difference in TME quality, tumor differentiation, lymphovascular invasion, nerve invasion, positive CRM, and number of total lymph nodes. After PSM, the length between the tumor and DRM was longer in the taTME group than the lapTME group (29.0 ± 7.8 *vs*. 25.8 ± 6.4 mm, *p* = 0.014), but was opposite before PSM. The number of metastatic lymph nodes was greater in the taTME group than lapTME group before PSM. However, after PSM, there was no significant difference between the two groups (*p* = 0.194). Both before and after PSM, there was less locally advanced RC in the taTME group than the lapTME group.

**Table 3:** Histopathological outcomes of the matched cohort.

Variable	taTME(n=61)	lapTME(n=61)	P value
Quality of TME, n (%)			0.570
Complete	53(86.9)	55(90.2)	
Nearly complete	8(13.1)	6(9.8)	
Length between tumor and DRM, mean ± SD, mm	29.0 ± 7.8	25.8 ± 6.4	0.014
Tumor differentiation, n (%)			0.093
Moderate	54(88.5)	47(77)	
Poor	7(11.5)	14(23)	
Lymphovascular invasion, n (%)	5(8.2)	7(11.5)	0.543
Nerve invasion, n (%)	5(8.2)	8(13.1)	0.379
Positive CRM, n (%)	1(1.6)	0(0)	1.000
Number of metastatic lymph nodes, median (IQR)	1.7(0-2)	2.6(0-3.5)	0.194
Number of total lymph nodes, median (IQR)	15.2(11-17.5)	16.5(12.5-20)	0.267
Pathology stage, n (%)			0.028
I	18(29.5)	6(9.8)	
II	15(24.6)	17(27.9)	
III	26(42.6)	37(60.7)	
IV	2(3.3)	1(1.6)	

taTME, transanal total mesorectal excision; lapTME, laparoscopic total mesorectal excision; TME, total mesorectal excision; DRM, distal resection margin; SD, standard deviation; CRM, circumferential resection margin; IQR, Interquartile range.

### Long-term outcomes

**Figure 2:**
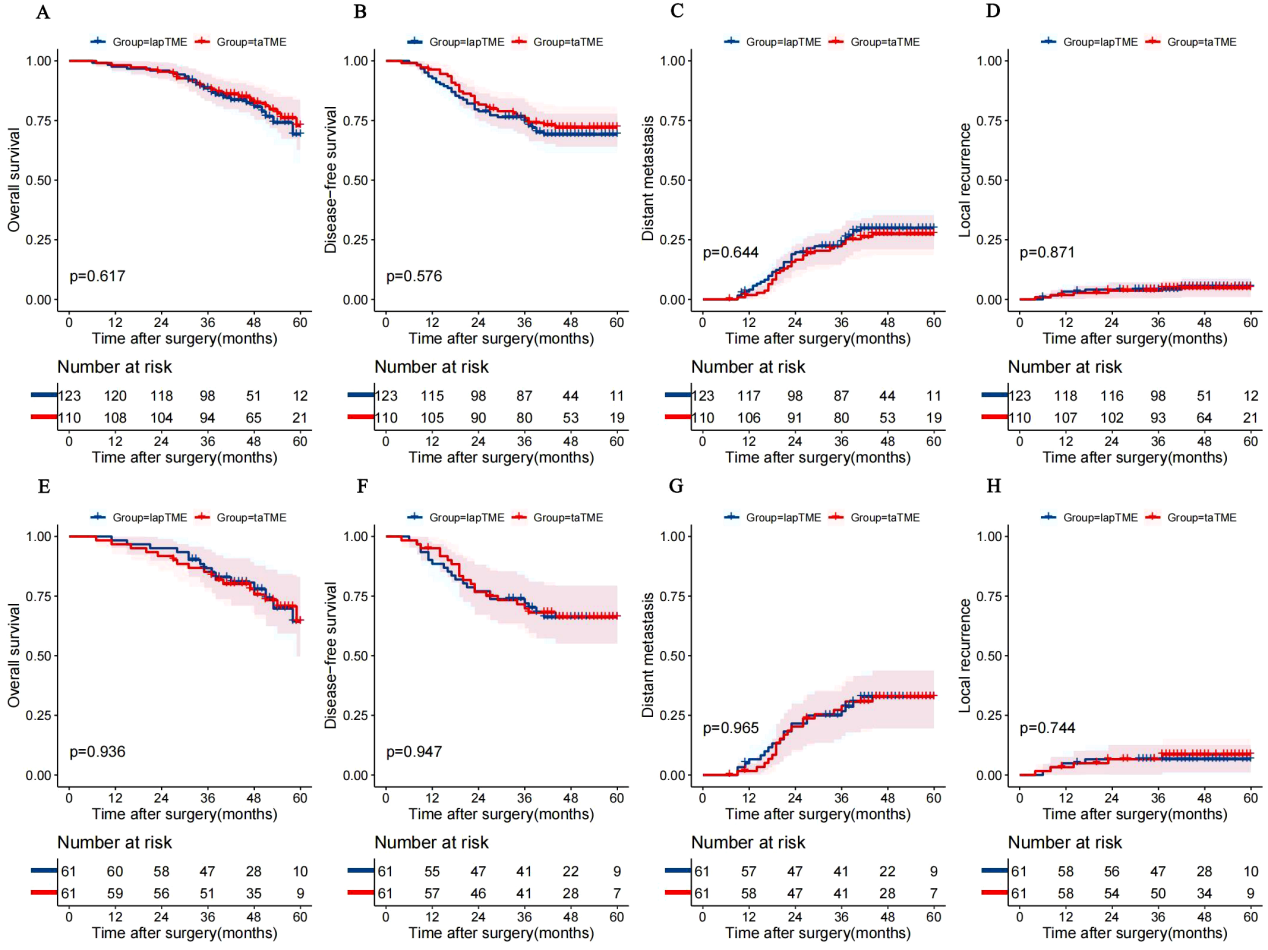
Kaplan–Meier curves of OS **(A)**, DFS **(B)**, DM **(C)** and LR **(D)** in the unmatched cohort. Kaplan–Meier curves of OS **(E)**, DFS **(F)**, DM **(G)** and LR **(H)** in the matched cohort. lapTME, laparoscopic total mesorectal resection; taTME, transanal total mesorectal resection; OS, overall survival; DFS, disease-free survival; DM, distant metastases; LR, local recurrence.

The long-term oncological outcomes of the two groups both before and after PSM are shown in **Supplementary Table S3**. After PSM, the median follow-up time was 51 and 46 months in the taTME and lapTME groups, respectively. Kaplan-Meier curves of OS, DFS, LR, and DM are presented in **Figure 2**.

Before PSM, there was no significant difference in the 3-year cumulative LR and DM rates between the taTME and lapTME groups (3.7% *vs*. 4.1%, *p* = 0.863; 23.3% *vs*. 24.1%, *p* = 0.888, respectively), which remained similar after PSM. Both before and after PSM, there were no significant differences in the cumulative 5-year LR and DM rates between the two groups (4.7% *vs*. 5.3%, *p* = 0.871; 27.3% *vs*. 29.8%, *p* = 0.644, respectively; 8.5% *vs*. 6.6%, *p* = 0.744; 32.8% *vs*. 32.7%, *p* = 0.965, respectively).

In the matched cohort, the 3-year OS and DFS rates were similar between the taTME and lapTME groups (85.2% *vs*. 86.7%, *p* = 0.808; 69.8% *vs*. 72%, *p* = 0.796, respectively), which remained in the unmatched cohort. In the unmatched and matched cohorts, there was no significant difference in 5-year DFS rates between two groups (72% *vs*. 69%, *p* = 0.576; 66.1% *vs*. 66.1%, *p* = 0.947, respectively). Importantly, the 5-year OS rate of the taTME group was 72.7% (95% CI, 61.9%–83.5%) before PSM, which was similar to that of the lapTME group (69.1%). After PSM, there was no significant difference in the 5-year OS rate between two groups (64.2% *vs*. 64.4%, *p* = 0.936).

### Cox proportional hazards regression analysis and subgroup analysis

The results of Cox proportional hazards regression analysis are shown in **Table 4**. Pathology stage, ASA greater than or equal to 3, and positive CRM were identified as risk factors for OS, while older age and pathology stage were related to DFS.

**Table 4:** Multivariate cox proportional hazards regression analysis.

Risk factors	Overall survival			Disease-free survival		
	HR	95%CI	P	HR	95%CI	P
Age	1.037	0.999-1.077	0.058	1.047	1.013-1.082	0.007
Pathology stage	4.337	2.155-8.730	0.001	4.613	2.269-9.381	0.001
Quality of TME				0.492	0.216-1.123	0.092
High ASA grade(≥3)	4.942	1.917-12.739	0.001			
Positive CRM	9.730	1.217-77.801	0.032			

HR, hazard ratio; CI, confidence intervals; TME, total mesorectal excision; ASA, American Society of Anesthesiologists; CRM, circumferential resection margin.

After PSM, subgroup analysis of the OS and DFS rates are presented in **Supplementary Figures S1**, **S2**, respectively. There was no significant difference in OS and DFS in terms of sex, BMI, clinical stage, comorbidity, ASA score, intraoperative complications, postoperative complications, anastomotic leakage, TME quality, tumor differentiation, nerve invasion, number of metastatic lymph nodes, and pT4 tumor stage.

## Discussion

In recent years, the oncological safety of taTME has gained momentous attention, primarily due to the suspension of this procedure in Norway. The results of current RCTs were highly expected. The findings of the present study revealed that taTME was associated with more nearly complete TME, higher rates of temporary stoma and handsewn anastomosis after PSM, which resulted from the early phase of the taTME learning curve and lower anastomosis from the anal verge. Importantly, taTME demonstrated comparable long-term oncological outcomes to lapTME.

RC surgery poses significant challenges owing to obesity and a narrow pelvis, which can substantially compromise the quality of conventional approaches (19). Several RCTs comparing taTME and lapTME are currently underway, but some meta-analyses have found that taTME provides reliable perioperative safety and histopathological security as compared to lapTME (20, 21). Similar to these findings, the results of this study showed comparable perioperative outcomes between the two techniques. Notably, taTME was associated with longer operative time and more estimated blood loss, which was attributed to a single-surgeon operative model, without increasing intraoperative complication rates. Radical resection remains the cornerstone of curative treatment for RC. The quality of such surgery can be evaluated through multiple pathological parameters, including mesorectal integrity, total lymph nodes detected, clear DRM, and clear CRM (22, 23), all of which are critical prognostic factors (24). Intraoperative frozen section analysis identified no positive DRM in our cohort. Although there was no significant difference in overall surgical quality between taTME and lapTME, our study revealed that taTME achieved longer DRM, which was identical to the results of Buan et al. (25). This finding further supports the technical reliability and oncological adequacy of taTME.

While the surgical quality of taTME is comparable to that of lapTME, the long-term oncological outcomes deserve greater attention. Maykel et al. reported that the 3-year OS and DFS rates of taTME were 86.6% and 82.6%, respectively (26). The International taTME Registry Collaborative, including 2803 patients who underwent taTME, reported 2-year DFS and OS rates of 77% and 92%, respectively (27). Kang et al. indicated that there was no significant difference in 5-year OS and DFS rates between taTME and lapTME (28). Consistent with these reports, no statistically significant difference was observed in 3- and 5-year OS and DFS rates between the two surgical procedures in this study. For low RC, anus preservation has become a common goal for both surgeons and patients. Some studies have shown that abdominal-perineal resection and taTME achieve similar oncological outcomes (29, 30). Robotic RC surgery offers stable manipulation, superior visualization, and better functional preservation of the pelvic autonomic nerve (31), while avoiding the “chopstick effect”. A multicenter RCT indicated that robotic surgery for mid-low RC can reduce the positive CRM rate, decrease the incidence of postoperative complications, and shorten the length of postoperative hospital stay as compared to lapTME (32). Simultaneously, it was associated with lower LR and improved DFS (33). Furthermore, robotic RC surgery and taTME demonstrated equivalent postoperative outcomes (10).

LR and DM directly impact survival time and quality of life, while imposing a heavier economic burden on society. Larsen et al. reported at least 10 diagnosed LRs in 110 patients who underwent taTME in Norway (34). A previous study by Gloor et al. reported that taTME achieved LR and DM rates of 5.4% and 26.7%, respectively (35). Lin et al. found that taTME was associated with a lower LR rate than lapTME (9.5% *vs*. 23.8%, *p* = 0.031), with no significant difference in the DM rate between the two groups (32.8% *vs*. 20.7%, *p* = 0.142) (36). A two-cohort study by Kang et al. revealed no significant difference in 3- and 5-year LR rates between taTME and lapTME (28). Our study found no statistically significant difference in LR and DM rates between the taTME and lapTME groups, which is consistent with previous reports. Although LR and DM rates had slightly increased after PSM, this may be primarily attributed to the learning curve associated with the taTME procedure. Consequently, high-quality structured training and overcoming the learning curve are essential to perform taTME surgery.

There were several limitations to this study that should be acknowledged. First, anal sphincter function, neurological function, and quality of life were not assessed. Second, the sample size was limited, particularly after PSM, resulting in reduced statistical power. Third, and most important, this was a single-center retrospective study and susceptible to selection bias. Fortunately, utilization of PSM ensured that baseline characteristics were similar between the two groups. Some studies have confirmed that there is no significant difference in postoperative anal function between taTME and lapTME (37, 38). Nevertheless, we believe that the better nerve sparing and preservation of the anal sphincter associated with taTME may have contributed to improved anal function. Therefore, multicenter RCTs are warranted to provide higher-level evidence for this aspect.

## Conclusion

The taTME group achieved comparable perioperative and long-term oncological outcomes as conventional lapTME. Nevertheless, as this was a single-center retrospective study, further RCTs are warranted to validate the efficacy and safety of this novel surgical technique.

## Data Availability

The original contributions presented in the study are included in the article/**Supplementary Material**. Further inquiries can be directed to the corresponding authors.
