# Supplementary material for: Long-term oncological outcomes of laparoscopic versus transanal total mesorectal excision for mid-low rectal cancer: a propensity score matching analysis
Source: Front Oncol. 2026 Feb 5;16:1715774. doi: 10.3389/fonc.2026.1715774 (PMC12916349; doi:10.3389/fonc.2026.1715774)
Supplement: Supplementary file 1 [file Table1.docx]

**Supplementary Tables**

Table S1. Operative details and clinical outcomes of the unmatched cohort.

|  | taTME(n=110) | lapTME(n=123) | P value |
| --- | --- | --- | --- |
| Operative time, mean ± SD, min | 278.0±74.7 | 242.7±24.2 | ＜0.001 |
| Estimated blood loss, median(95% CI), ml | 100(113.0-142.0) | 100(95.4-114.6) | 0.011 |
| Enterostomy, n (%) |  |  | ＜0.001 |
| Yes | 50(45.5) | 23(18.7) |  |
| No | 60(54.5) | 100(81.3) |  |
| Intraoperative complications, n (%) | 3(2.7) | 4(3.3) | 1.000 |
| Type of anastomosis, n (%) |  |  | ＜0.001 |
| Stapled | 88(80.0) | 123(100.0) |  |
| Handsewn | 22(20.0) | 0(0) |  |
| Postoperative complications, n (%) | 25(22.7) | 25(20.3) | 0.656 |
| Anastomotic leak, n (%) | 11(10.0) | 8(6.5) | 0.330 |
| Clavien-Dindo grade 3+ complications, n (%) | 4(3.6) | 3(2.4) | 0.710 |

taTME, transanal total mesorectal excision; lapTME, laparoscopic total mesorectal excision; SD, standard deviation; CI, confidence intervals.

Table S2. Histopathological outcomes of the unmatched cohort.

|  | taTME(n=110) | lapTME(n=123) | P value |
| --- | --- | --- | --- |
| Quality of TME, n (%) |  |  | 0.469 |
| Complete | 98(89.1) | 113(91.9) |  |
| Nearly complete | 12(10.9) | 10(8.1) |  |
| Length between tumor and DRM, mean ± SD, mm | 25.8±7.8 | 28.3±7.3 | 0.010 |
| Tumor differentiation, n (%) |  |  | 0.068 |
| Moderate | 99(90.0) | 102(82.9) |  |
| Poor | 10(9.1) | 21(17.1) |  |
| PCR | 1(0.9) | 0(0) |  |
| Lymphovascular invasion, n (%) | 13(11.8) | 15(12.4) | 0.930 |
| Nerve invasion, n (%) | 12(10.9) | 18(14.6) | 0.397 |
| Positive CRM, n (%) | 1(0.9) | 1(0.8) | 1.000 |
| Number of metastatic lymph nodes, median (IQR) | 0(0.0-1.0) | 1.0(0.0-4.0) | 0.003 |
| Number of total lymph nodes, median (IQR) | 13.0(11.0-17.0) | 15.0(12.0-19.0) | 0.070 |
| Pathology stage, n (%) |  |  | ＜0.001 |
| 0 + PCR | 1(0.9) | 0(0) |  |
| I | 38(34.5) | 9(7.3) |  |
| II | 31(28.2) | 36(29.3) |  |
| III | 38(34.5) | 76(61.8) |  |
| Ⅳ | 2(1.8) | 2(1.6) |  |

taTME, transanal total mesorectal excision; lapTME, laparoscopic total mesorectal excision; TME, total mesorectal excision; DRM, distal resection margin; SD, standard deviation; PCR, pathologic complete response; CRM, circumferential resection margin; IQR, interquartile range.

Table S3. Oncology outcomes

|  | Before PSM | |  | After PSM | |  |
| --- | --- | --- | --- | --- | --- | --- |
|  | taTME | lapTME | *P* value | taTME | lapTME | *P* value |
| Median follow-up: months (IQR) | 51.5  (43.0-58.0) | 46.0  (38.0-53.0) |  | 51  (41.5-57.5) | 46  (37-55) |  |
| 3-Year OS rate in percent (95% CI) | 88.9%  (83%-94.8%) | 88.5%  (82.8%-94.2%) | 0.912 | 85.2%  (76.2%-94.2%) | 86.7%  (78.1%-95.3%) | 0.808 |
| 3-Year DFS rate in percent (95% CI) | 76%  (68%-84%) | 74.6%  (66.9%-82.3%) | 0.809 | 69.8%  (58.1%-81.5%) | 72%  (60.7%-83.3%) | 0.796 |
| 3-Year cumulative LR rate in percent (95% CI) | 3.7%  (0.2%-7.2%) | 4.1%  (0.6%-7.6%) | 0.863 | 6.6%  (0.2%-13.0%) | 6.6%  (0.4%-12.8%) | 0.990 |
| 3-Year cumulative metastasis rate in percent (95% CI) | 23.3%  (15.3%-31.3%) | 24.1%  (16.5%-31.7%) | 0.888 | 29%  (17.4%-40.6%) | 26.8%  (15.5%-38.1%) | 0.784 |
| 5-Year OS rate in percent (95% CI) | 72.7%  (61.9%-83.5%) | 69.1%  (55.8%-82.4%) | 0.617 | 64.2%  (47.7%-80.7%) | 64.4%  (47.4%-81.4%) | 0.936 |
| 5-Year DFS rate in percent (95% CI) | 72%  (63.5%-80.5%) | 69%  (60.7%-77.3%) | 0.576 | 66.1%  (54%-78.2%) | 66.1%  (53.9%-78.3%) | 0.947 |
| 5-Year cumulative LR rate in percent (95% CI) | 4.7%  (0.7%-8.7%) | 5.3%  (1.1%-9.5%) | 0.871 | 8.5%  (1.3%-15.7%) | 6.6%  (0.4%-12.8%) | 0.744 |
| 5-Year cumulative metastasis rate in percent (95% CI) | 27.3%  (18.8%-35.8%) | 29.8%  (21.5%-38.1%) | 0.644 | 32.8%  (20.7%-44.9%) | 32.7%  (20.5%-44.9%) | 0.965 |

PSM, propensity score matching; taTME, transanal total mesorectal excision; lapTME, laparoscopic total mesorectal excision; IQR, interquartile range; OS, overall survival; CI, confidence intervals; DFS, disease-free survival; LR, local recurrence.
