# Supplementary material for: Long-term oncological outcomes of laparoscopic versus transanal total mesorectal excision for mid-low rectal cancer: a propensity score matching analysis
Source: Front Oncol. 2026 Feb 5;16:1715774. doi: 10.3389/fonc.2026.1715774 (PMC12916349; doi:10.3389/fonc.2026.1715774)
Supplement: Supplementary file 2 [file DataSheet1.docx]

**Supplementary Figures**

Figure S1. Subgroup analysis of overall survival


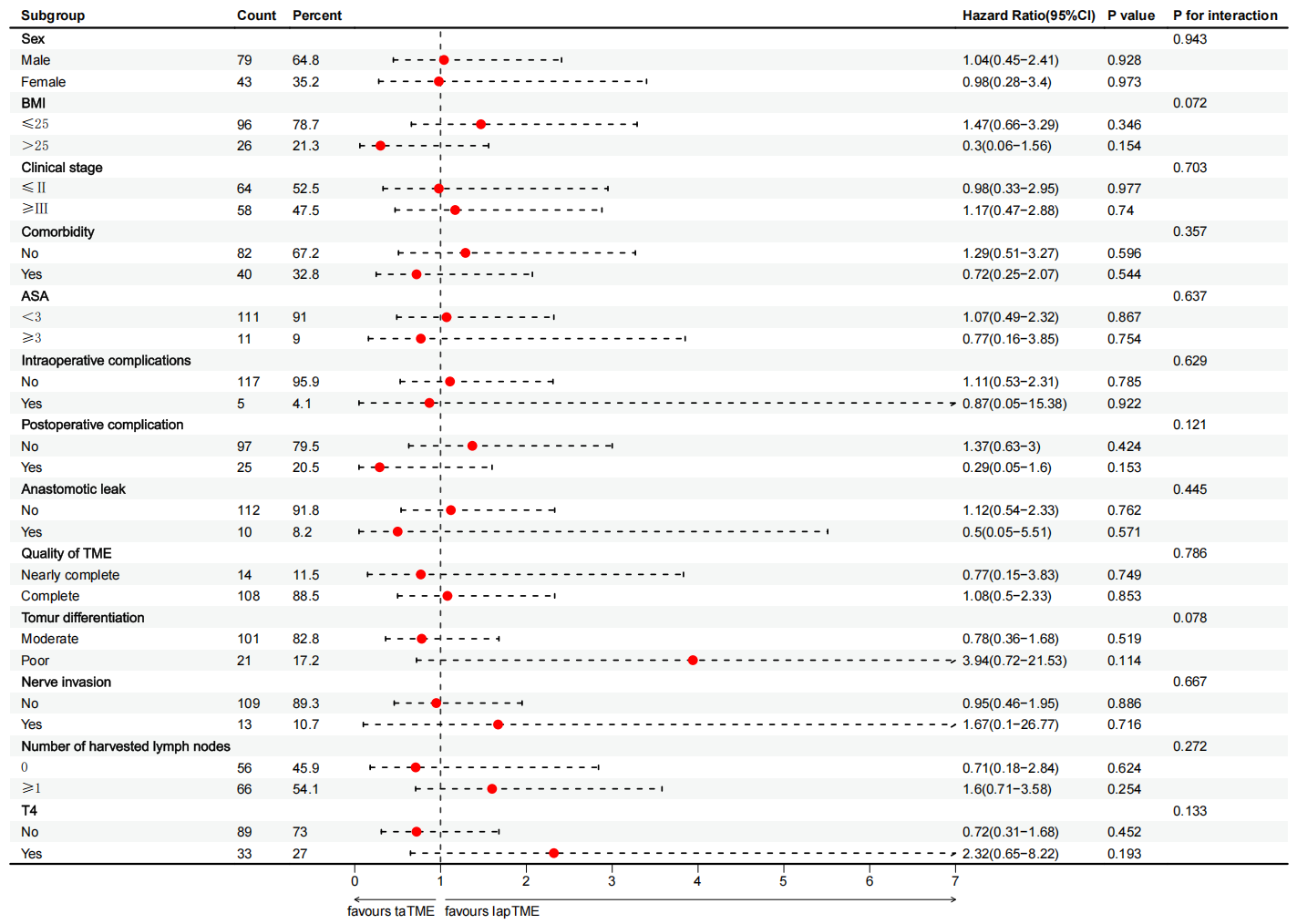


BMI Body mass index, ASA American Society of Anesthesiologists, TME total mesorectal excision.

Figure S2. Subgroup analysis of disease-free survival


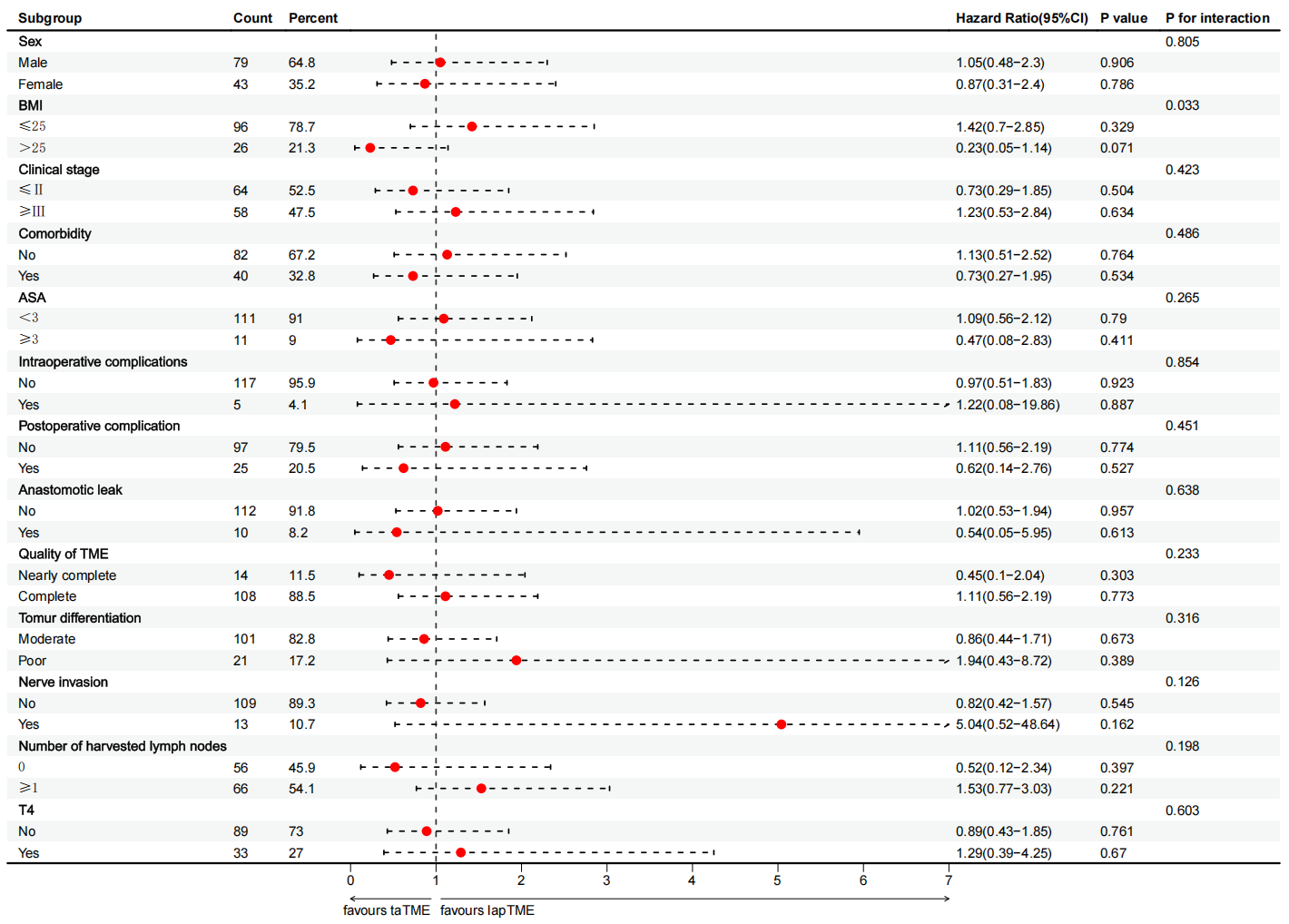


BMI Body mass index, ASA American Society of Anesthesiologists, TME total mesorectal excision.
